# Knowledge, attitudes, and practice of community pharmacists towards providing counseling on vitamins, and nutritional supplements in Saudi Arabia

**DOI:** 10.3934/publichealth.2020054

**Published:** 2020-09-15

**Authors:** Ali Alshahrani

**Affiliations:** Clinical Pharmacy, Pharmacy college, Taif University, Taif, Saudi Arabia

**Keywords:** knowledge, attitudes, practice, community pharmacists, counseling, vitamins, nutritional supplements, Saudi Arabia

## Abstract

**Background:**

Pharmacists play an important role in the healthcare system and have a greater impact on the outcome of public health programs. Patients who seek nutrition and vitamin advice presume the role of community pharmacists including the recommendation of effective vitamin and nutritional products.

**Objective:**

To determine the knowledge, attitudes, and practice of community pharmacists towards providing counseling on vitamins and nutritional supplements in Saudi Arabia.

**Setting:**

All community pharmacies in all cities in Saudi Arabia.

**Methods:**

A cross-sectional study methodology. The study was conducted between September 2019 and April 2020. Google survey was used to collect the samples from community pharmacists across Saudi Arabia.

**Main outcome measure:**

Main outcome measure knowledge, attitudes, and practice of community pharmacists towards providing counseling on vitamins and nutritional supplements.

**Results:**

A total of 1199 questionnaires were distributed electronically. Only 1041 were obtained to representing an 86.8% success rate. A significant majority of the respondents in this study were males (98.7%) and aged between 23–34 years (80.7%). Most of the community pharmacists in this study were non-Saudi residents 96.4%. Only 24.0% had certification from the Saudi National Board. Daily, the majority of the pharmacists attended to an estimated 100–200 patients 62.3% with only 22.7% attending to more than 200 customers per day. Most of the community pharmacists 86.4% have received a form of training on vitamins and nutritional supplements. Most of the community pharmacists counsel their patients about the side effects of vitamins and nutritional supplements.

**Conclusion:**

Community pharmacists in Saudi Arabia have adequate knowledge and a positive attitude about vitamins and nutritional supplements. Our findings indicate that training courses are needed to improve pharmacists' communication skills to play more roles in counseling and enhancing their practice.

## 1. Introduction

The last few decades have been characterized by increased recognition of the role that community pharmacists play in patient care [1]. A study by Quasim and Bayunus [2] revealed that community pharmacists received visits from patients 35 times a year compared to the four times a year a primary care physician was visited. While the general medical practitioners have traditionally been recognized as the gatekeepers of primary care, studies show that community pharmacists receive an estimated 12 to 15 consultations each year [1]. Community pharmacists are strategically positioned to play a central role in care delivery; moreover, Kayyali [3] pointed out that community pharmacists are the largest and most accessible healthcare professional since they are located at the frontline of care delivery. Pharmacists play an important role in the healthcare system and have a greater impact on the outcome of public health programs. Community pharmacist has traditionally played a critical role in patient training [4]. However, numerous studies have pointed to deficiencies in pharmacist-patient communication regarding health products and nutritional choices. Some studies have suggested the need for effective training to promote improved healthcare outcomes. According to Hadi et al. [5],[6], patients who seek nutrition and vitamin advice presume the role of community pharmacists include recommendation of effective vitamin and nutritional products. However, the professional responsibilities of pharmacists with respect to product and advice on nutrition is not well established.

Besides, the nature of therapeutic conditions in vitamins and nutritional deficiency cases means that the success of complementary medication depends on findings that right therapy which varies from an individual to individual [7]. This implies that community pharmacists must have knowledge about vitamin and nutritional supplement products to effectively advise the consumers on vitamins, and nutrition, deficiencies treatment. Consequently, they must have the right attitude and appropriate counseling skills to effectively dispense their duties. Prior studies have shown that consumers routinely ask their pharmacist for information about vitamins and nutritional choices and seek advice on these products as part of the pharmacy practice [8],[9]. A previous survey in Australia found that an estimated 87% of the consumers expect pharmacists to provide them with sufficient information about nutrition and vitamin product efficacy. 92% of those who were surveyed in this study pointed out that they also expected to receive safety information regarding these products [10]. Most of the vitamin and nutrition supplement consumers believe that the role of pharmacists is to provide recommendations about effective vitamin and nutrition supplement products and reliably detect and restrain interactions between the conventional medicines and supplementary nutritional products [7].

Despite the high-level expectation about community pharmacists by vitamin and nutritional supplement seekers, most of the pharmacists rate their knowledge about CAMs as inadequate. A study conducted by Abadel [11] found that most community pharmacists do not feel confident in answering patient questions on dietary and nutritional choices. Numerous studies have also illustrated the lack of knowledge or inadequate education in supplement products among pharmacists [12]–[14]. The lack of knowledge incapacitates pharmacists to provide informative consultation on nutritional and vitamin choices to the consumers. The lack of counseling skills has also been cited as one of the primary challenges that many pharmacists face while providing care [15],[16]. To our knowledge, there is paucity in studies considering pharmacists' knowledge, attitudes, and practice of community pharmacists in Saudi Arabia towards providing counseling on vitamins and nutritional products. Thus, this study aimed to study knowledge, attitudes, and practice of community pharmacists towards providing counseling on vitamins and nutrition deficiencies treatment in Saudi Arabia. The objective of this study was to determine the knowledge, attitudes, and practice of community pharmacists towards providing counselling on vitamins and nutrition deficiencies treatment in Saudi Arabia.

## 2. Materials and methods

### 2.1. Study design and selection criteria

A cross-sectional study design was adopted to determine the knowledge, attitudes, and practice of community pharmacists towards providing counseling on vitamins and nutrition deficiencies treatment in Saudi Arabia. The study was conducted between September 2019 and April 2020.

The study targeted registered community pharmacists working directly with pharmaceutical product consumers in the country. They must be registered as pharmacist and work in community pharmacies in the country. The pharmacist must have at least a bachelor's degree certification or higher degree and willing to participate in the study.

### 2.2. Study procedure

The study processes began with the construction of survey instruments that were designed to capture data on the knowledge, attitudes, and practice of community pharmacists towards providing counseling on vitamins and nutritional supplements. First, all survey questions were obtained from relevant literature. Then, field experts selected questions to determine the knowledge, attitudes, and practice of community pharmacists towards providing counseling on vitamins and nutritional supplements in Saudi Arabia. To validate the survey content, we conducted a pilot study. Finally, we used Cronbach's alpha coefficient test to measure the internal consistency.

The survey instruments were organized into three sections. The first section captured the demographic characteristics of the targeted population—age, gender, level of education, years of practice, area of residence, and practice setting. The second section captured the pharmacists' knowledge about vitamins and nutritional deficiencies. A five-point Likert scale ranging from 1 strongly agree to 5 strongly disagree was used to capture the knowledge of the community pharmacists. The last section to covered professional practice, attitudes, and beliefs towards counseling by the community pharmacists on nutritional and supplementary products. Practice was measured by the frequency of counselling consumers while dispensing vitamins and nutritional supplements, ability to make follow up after dispensing vitamins and nutritional supplements, frequency of customer demand for more information, and the materials relied on when making decision on which vitamins and supplements to dispense.

The survey instrument was then entered into google survey instrument and distributed to the target population. It was expected that each interview will take an average of 10 to 15 minutes. To increase the rates of participation in the study, the researcher offered a clear explanation of the purpose of the study and the potential benefits associated with the results of the study to pharmacy as a profession as well as the benefits to the patient and the health sector in general.

### 2.3. Statistical methods

The data collected was cleaned, organized, and entered in the Statistical Package for Social Sciences SPSS version 25.0. The demographic characteristics of the sampled population was analyzed and grouped based on their descriptive statistics. To determine the pharmacists' attitudes and practices towards providing counseling on vitamins, and nutrition, deficiencies treatment, a Kruskal Wallis test followed by pairwise comparison test was conducted. A Kruskal Wallis Test is a non-parametric alternative to one-way ANOVA test which is used to test for differences between groups when the dependent variables are ordinal. A *p-value* of 0.5 was considered statistically significant.

### 2.4. Ethical consideration

An ethical approval was obtained from the authority responsible for pharmacy research and ethics in the University board. In addition, a written consent form was provided electronically where the respondents had to agree to participating into the research before filling the questionnaires. The consent form explained the purpose of the study, guaranteed data safety, and explained how the data will be used in the study. Imperatively, no personally identifiable data will be used in the study.

## 3. Results

### 3.1. Reliability and consistency

Cronbach's alpha test was 0.84 on the seven questions regarding the practice of Pharmacists in Saudi Arabia measured on a Likert scale. The alpha test value shows that the survey has a good internal consistency.

### 3.2. Demographic characteristics

A total of 1199 questionnaires were distributed electronically. Of this, only 1041 were responded to representing an 86.8% success rate. A significant majority of the respondents in this study (80.7%) were young pharmacists in their early years of their career (23–34 years), 17.9% were within the age bracket of 35 and 44 years while 1.3% of the respondents were between the age of 45–54 years. Majority of the respondents were males (98.7%) with women taking a minority status in the community pharmacy circles (1.3%). Most of respondents were non-Saudi residents (96.4%) while only 3.6% of the sampled population were Saudi residents. A significant majority of the community pharmacists in this study were married (78.4%), 0.4% had been divorced, while 21.2% were single. Most of the community pharmacist surveyed in this study were working from cities (91.0%) with only a small proportion working in the villages (9.0%). The western and central region of Saudi Arabia represented the largest block of the community pharmacists in this study (35.6% and 24.0% respectively). The southern region of the country accounted for 19.9 % of the total surveyed population, while the rest of the regions were distributed almost equally (9.4% eastern region, 11.0% for the Northern region). To a large extent, the samples collected represented the opinion of pharmacists on all the blocks of the country. Majority of the pharmacists working in the country have a minimum qualification of a bachelor's degree, (88.9%). Most of the pharmacists surveyed in this study described themselves as pharmacists (75.6%) with 23.2% considering themselves as senior pharmacists. A small number of community pharmacist surveyed in this study did not have Saudi board certification, accounting for 76.0% of the total population. Only 24.0% had certification from the board. For years of practice, 47.5% of the surveyed population had 6–10 years of experience as pharmacists, 26.2% had 2–5 years of experience, 23.3% of the population had over ten years of experience as community pharmacists, while 3.2% were under the two-year experience. Daily, the majority of the pharmacists attended to an estimated 100–200 patients (62.3%) with only 22.7% attending to more than 200 customers per day. At least 15.0% of the surveyed population attended to less than 100 customers in a single day (Table 1).

### 3.3. Pharmacists knowledge about vitamins and nutrition deficiencies treatment in Saudi Arabia

**Figure 1.**
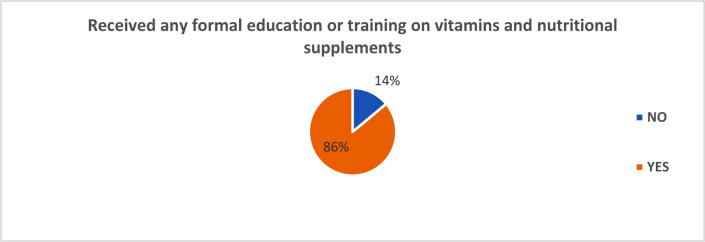
Pie chart of educational training on vitamins and nutritional supplements.

To test the knowledge of community pharmacist about vitamins and nutritional deficiency treatment in the Kingdom of Saudi Arabia, the researcher obtained responses on the pharmacists training on vitamins and nutritional substance, the most frequent consumers of vitamins and nutritional supplements, the top five vitamins and nutritional substances in their point of sale, and the most common uses of vitamins and nutritional products [15]. When asked whether they had received any form of training with respect to vitamins and nutritional supplements, 86.4% agreed to have received a form of training in vitamins and nutritional supplements, 13.6% had not received any form to training concerning nutritional supplements and vitamins. Figure 1 captures the graphical representation of the data.

**Table 1.** Demographic Characteristics of the Study Population.

ITEMS	Descriptive	Frequency	Percent
Age	23–34 years	840	80.7
	35–44 years	186	17.9
	45–54 years	14	1.3
	55–60 years	1	0.1
Gender	Female	14	1.3
	Male	1027	98.7
Nationality	Non-Saudi	1004	96.4
	Saudi	37	3.6
Marital Status	Divorced	4	0.4
	Married	816	78.4
	Single	221	21.2
Work Location	In a city	947	79
	In a village	94	7.8
Region	Central region	250	24.0
	Eastern region	98	9.4
	Northern region	115	11.0
	Southern region	207	19.9
	Western region	371	35.6
Education; Experience; Service
Qualification	Diploma	9	0.9
	Bachelor	925	88.9
	Master	9	0.9
	Pharm.D	97	9.3
	PhD	1	0.1
Present professional level	Technician	1	0.1
	Pharmacist	787	75.6
	Senior pharmacist	241	23.2
	Consultant pharmacist	12	1.2
Board-certification	No	791	76.0
	Yes	250	24.0
Work Setting	Chain pharmacy	1036	99.5
	Independent pharmacy	5	0.5
Experience	< 2 years	33	3.2
	>10 years	241	23.2
	2–5 years	273	26.2
	6–10 years	494	47.5
Patients/customers Served Daily	≤100	156	15.0
	100–200	649	22.7
	≥200	236	62.3

The responded were asked to choose five of the most commonly consumed multivitamins from a list of 36 vitamins and nutritional supplements as follows Vit C., Vit E, Vit A, Fish oil, Amino acids, Antioxidants, Beta-carotene, Bee-pollen, Black cohosh, Citrus Aurantium, Creatine, Cyanocobalamin, Echinacea, Eucalyptus, Fibers, Folate Garlic, Dietary supplement, Ginkgo biloba, Ginseng, Hawthorn, Horse chestnut seed, Kava-kava, Lecithin, Macronutrients, Pyruvate, Saw palmetto, Senna, Omega-3, Shark cartilage, St John's wort, Valerian root, Whey protein, Protein powder, Multivitamins, Caffeine, Glutamine, and Multimineral. From this list, the respondents produced a list characterized by hundreds of combinations based on the characteristics of their point of sale. Specifically, 452 combinations from the selected drugs were products with some gaining the most frequency. Multivitamins, fish oil, omega-3 antioxidants, and vitamin C appeared in most of the combination. The data was then further sorted to identify similarities in combination from the community pharmacist. The following diagrams capture the most popular combination from the list of 452 produced by the respondents. From the table, a combination of Multivitamins, Vit C., Vit. D., Fish oil, Omega-3 was produced 94 times accounting 7.8% of the total combinations produced. The distribution of the various combinations generated is shown below (Table 2). (no specific order was chosen in the presentation of the combinations).

**Table 2.** Knowledge on the Top 5 vitamins and nutritional supplements.

No.	Top 5 Vitamins and Nutritional Supplements	Frequency	Percent
1.	Multivitamins, Vit C., Vit. D., Fish oil, Omega-3	94	7.8
2.	Multivitamins, Vit C., Vit. D., Ginseng, Omega-3	37	3.1
3.	Multivitamins, Vit C., Vit E., Vit. D., Omega-3	35	2.9
4.	Multivitamins, Vit C., Vit. D., Cyanocobalamin, Omega-3	31	2.6
5.	Multivitamins, Vit C., Vit. D., Fish oil, Ginseng	19	1.6

(not every respondent produced a list of five items, others produced 3).

To identify the most common users of vitamins and multivitamins, the pharmacists were asked to choose a combination from a list of 18 possible users as follows, Anemia, Blood disorder, Boosting immunity, Cold/Flu, Diabetes, Elderly situations, General health, Hair, skin and nail condition, Hypertension, Loss of appetite, Nutritional deficiencies, Pregnancy and lactation Prophylactic antioxidant, Reducing cholesterol, Smoking, URTI/UTI , and Weight gain/loss.

From this list, the respondents produced a combination of sixty possible users based on the usage of vitamins and nutritional supplements from their point of sale. Children, Adult men, Elderly, Pregnant or lactating women, Patients with Chronic Diseases were identified as the most common users of vitamins and nutritional supplements accounting for 13.2% of the total population surveyed. Imperatively, adult men, the elderly, and children featured predominantly in all the combination produced from the provided options (Table 3).

**Table 3.** Knowledge on Consumer profile.

No.	Users Profile	Frequency	Percent
1.	Children, Adult men, Elderly, Pregnant or lactating women, Patients with Chronic Diseases	158	13.2
2.	Children, Adult men, Adult women, Elderly, Pregnant or lactating women, Patients with Chronic Diseases	128	10.7
3.	Adult men	63	5.3
4.	Children, Elderly, Pregnant or lactating women, Patients with Chronic Diseases	53	4.4
5.	Children, Elderly, Pregnant or lactating women	43	3.6
6.	Elderly, Pregnant or lactating women, Patients with Chronic Diseases	41	3.4
7.	Adult men, Adult women, Pregnant or lactating women	37	3.1
8.	Children, Pregnant or lactating women, Patients with Chronic Diseases	36	3
9.	Adult men, Pregnant or lactating women	34	2.8
10.	Adult men, Adult women	32	2.7

### 3.4. Practice

The practice of community pharmacist in this study was assessed by obtaining responses concerning frequency of counselling consumers while dispensing vitamins and nutritional supplements, ability to make follow up after dispensing vitamins and nutritional supplements, frequency of customer demand for more information, and the materials relied on when making the decision on which vitamins and supplements to dispense. The practice of counselling is a complex process; however, these activities were identified as the key elements in the practice of community pharmacist while executing their duties.

When asked how often they counsel patients and consumers about the side effects of vitamins and nutritional supplements consumption, a descriptive analysis of the sample resulted in a mean of 4.19 with a statistic skew of −0.811. The histogram output was skewed to the right, indicating that most of the responses ranged from quite often to always. These results indicated that most of the community pharmacist counsel their patients and consumers about the side effects of vitamins and nutritional supplements (Table 4).

**Table 4.** Frequency of Counselling.

		Frequency	Percent
How frequently do you counsel patients/consumers about the side effects of vitamins and nutritional supplements?	1 Never	6	0.58
	2 Rarely	16	1.54
	3 Often	167	16.04
	4 Mostly	436	41.88
	5 Always	416	39.96
	Total	1041	100

**Figure 2.**
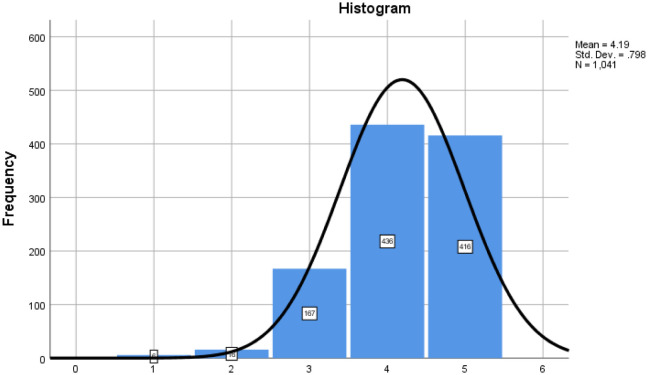
Patient Follow Up (Do you follow up with the patients who continually consume vitamins and nutritional supplements to record any side effects or beneficial effects?)

**Figure 3.**
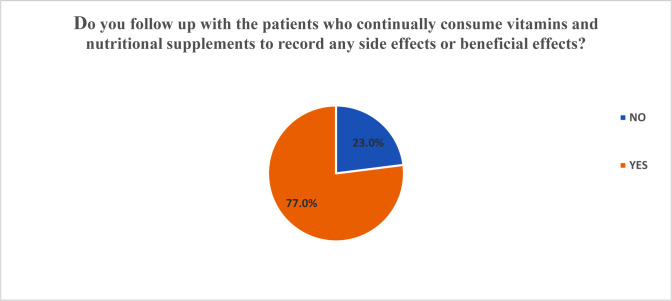
Pie chart of follow ups on patients for record fo side effects or benefitila effects.

As to whether the respondents made follow-ups after dispensing vitamins and supplements to patients and consumers, a significant majority of the surveyed population, 66.72%, agreed to conducting follow-ups (Figure 2). An estimated 20% of the surveyed community pharmacist in the country did not make follow up after dispensing vitamins and nutritional supplements. The diagram below captures the results of the analysis (Figure 3).

**Figure 4.**
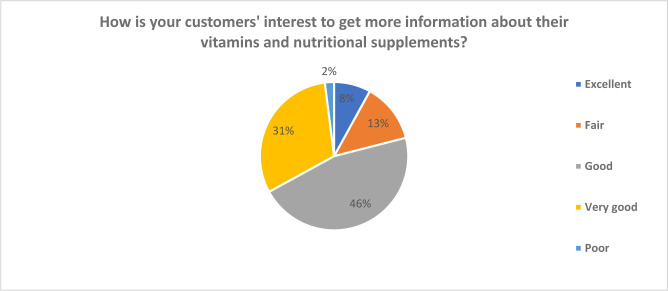
Customers' interest to get more information about their vitamins and nutritional supplements.

As to whether the consumers are interested in obtaining information about the nutritional supplements and vitamins dispensed in community hospitals, it was established that the largest population of consumers were interested in obtaining more information about the nature of the products being consumed. The data was distributed between very good 27.36% and good 39.70% indicating that over 75% of the consumers attending community pharmacist are interested in obtaining data about the nature of vitamins and nutritional supplements dispensed. (Figure 4)

Equally important in determining the ability of community pharmacists to make informed counselling decisions during practice are the facts that influence their decision making when prescribing, recommending or dispensing vitamins and nutritional supplements. A percentile analysis of the responses indicated that a combination of availability, effectiveness and cost were the leading factors considered when making recommendations, prescription or dispensing vitamins and nutritional supplements among Saudi Arabian community pharmacists (Table 5).

**Table 5.** Top 5 Factors considered for vitamins and nutritional supplement recommendation.

Factor	Percent
Both Effectiveness and cost	28.5
Effectiveness	20.7
Both effectiveness and cost, ADR	8.2
Effectiveness, ADR	6.3
Both effectiveness and cost, availability	4.4

## 4. Discussion

The aim of this study was to determine the knowledge, attitudes, and practice of community pharmacists towards providing counselling on vitamins and nutrition deficiencies treatment in Saudi Arabia. Today, it is widely acknowledged that community pharmacist is more likely to prescribe medication a shift from their traditional role of dispensing drugs. Another important trend in contemporary Saudi is the increasing consumption of vitamins and supplementary products. The Saudi population is traditionally known for its use of alternative medicines; vitamins and nutritional supplements are among the most popularly consumed medical products in Saudi today [11],[18]. The pharmaceutical demands of society mean that community pharmacists must demonstrate efficacy in the knowledge, competence in the execution of the duties and the right attitudes towards counselling while executing their duties. Yet, despite the high-level expectation about community pharmacists by vitamin and nutritional supplement seekers, most of the pharmacists rate their knowledge about CAMs as inadequate. The number of studies demonstrating this fact is myriad. The most popular of these studies include those conducted by Abadel [11], who found that most of the community pharmacists do not feel confident in answering patient questions on vitamin and nutritional choices. Numerous studies have also illustrated the lack of knowledge or inadequate education in supplement products among pharmacists [12]–[14]. The lack of knowledge incapacitates pharmacists to provide informative consultation on nutritional and vitamin choices to the consumers. Besides, the lack of counselling skills has also been cited as one of the primary challenges that many pharmacists face while providing care. This study closes this gap by assessing how community pharmacists in Saudi Arabia are prepared to handle the complex demand of the Saudi population concerning vitamins and nutritional supplement use.

Demographically, the sampled community pharmacists were composed of expatriates suggesting that a large number of the community pharmacists in the country were experts from other countries. Also, most of the community pharmacists were males which was consistent with the tradition and regulations of the country in relation to community pharmacists' services. The least qualification for most of the community pharmacists surveyed for this study was the bachelor's degree.

### 4.1. Knowledge and the ability to confidently offer vitamins and nutritional counselling to patients

The pharmacists surveyed in this study were extensively informed about the most common forms of vitamins and nutritional supplements demands of the Saudi Arabian population. For example, it was established that out of a list of 36 vitamins and nutritional supplements Vit C., Vit E, Vit A, Fish oil, Amino acids, Antioxidants, Beta-carotene, Bee-pollen, Black cohosh, Citrus Aurantia, Creatine, Cyanocobalamin, Echinacea, Eucalyptus, Fibers, Folate Garlic, Dietary supplement, Ginkgo biloba, Ginseng, Hawthorn, Horse chestnut seed, Kava-kava, Lecithin, Macronutrients, Pyruvate, Saw palmetto, Senna, Omega-3, Shark cartilage, St John's wort, Valerian root, Whey protein, Protein powder, Multivitamins, Caffeine, Glutamine, and Multimineral. Multivitamins, fish oil, omega-3 antioxidants, and vitamin C appeared in most of the combination. The data was then further sorted to identify similarities in combination from the community pharmacist and a combination of Multivitamins, Vit C., Vit. D., Fish oil, Omega-3 was produced 94 times accounting for 7.8% of the total combinations produced. This result demonstrated knowledge not just of the available vitamins and nutritional supplements but also the nature of products consumed within the country. Also, the sample of community pharmacists surveyed in this study was the most common users of vitamins and nutritional supplements. From a list of items designed specifically to test the knowledge of the community pharmacists on the type of consumers of vitamins and nutritional products, the respondents produced a combination of sixty possible users. Children, Adult men, Elderly, Pregnant or lactating women, Patients with Chronic Diseases were identified as the most common users of vitamins and nutritional supplements accounting for 13.2% of the total population surveyed. Imperatively, adult men, the elderly, and children featured predominantly in all the combination produced from the provided options.

To determine whether the knowledge possessed by the surveyed sample was sufficient to adopt or reject the first hypothesis raised in this study, comparative analysis in the form of Friedman test—a non-parametric alternative to one-way ANOVA test which is used to test for differences between groups when the dependent variables are ordinal was used. A *p-value* of 0.5 indicated sufficiency in knowledge to support the hypothesis that Community pharmacists in the country possess adequate knowledge to confidently offer vitamin and nutrition counselling to patients in the country. Knowledge is cited as one of the primary tenets determining the capacities of community pharmacists. Community pharmacists are expected to be aware of a wide range of pharmaceutical products, side effects of various products, remedies to the side effects, the reaction of drugs in relation to other products, as well as the nature of consumers of their products. Knowledge is a strategic resource in community pharmacy has been established in many studies. Shilbayeh [15] found that knowledge about pharmaceutical products was central to the effective dispensing of pharmaceutical products among community pharmacist in Palestine. In this study, the community pharmacists surveyed demonstrated sufficient knowledge on the consumers, vitamins and nutrients supplements and counselling.

### 4.2. Attitude and the practice towards counselling

As identified, previous practice in this study was assessed through community pharmacists' ability to counsel consumers, while dispensing vitamins and nutritional supplements, ability to make follow-ups after dispensing vitamins and nutritional supplements, frequency of customer demand for more information, and the materials relied on when making the decision on which vitamins and supplements to dispense [19]–[22]. In relation to the practice of counselling, a mean of 4.19 with a statistical skew of −0.811 was recorded. These results indicated that most of the community pharmacist counsel their patients and consumers about the side effects of vitamins and nutritional supplements. As to whether the respondents made follow-ups after dispensing vitamins and supplements to patients and consumers, a significant majority of the surveyed population, 66.72%, agreed to make follow-ups. An estimated 20% of the surveyed community pharmacist in the country did not make follow up after dispensing vitamins and nutritional supplements Equally important in determining the ability of community pharmacist to make informed counselling decisions during practice what the facts that influence their decision making when prescribing, recommending or dispensing vitamins and nutritional supplements. A percentile analysis of the responses indicated that a combination of availability, effectiveness and cost were the leading factors considered when making recommendations, prescription or dispensing vitamins and nutritional supplements among Saudi Arabian community pharmacists.

The paper set out to determine whether the community pharmacists surveyed in the country possessed the right attitude towards the practice of counselling for vitamins and nutritional supplements by comparing how attitudes relate to counselling practice. The results of the study demonstrated that community pharmacists in Saudi Arabia possess positive attitudes and practice towards providing counselling on vitamins, and nutrition, deficiencies treatment. The results of this study painted a positive picture with respect to the practice of counselling among community pharmacists when dispensing, recommending or prescribing vitamins and nutritional supplements. The results of this study are consistent with previous studies in the region with respect to the practice of community pharmacist in dispensing vitamins and nutritional supplements. A study conducted by Ghosn, Addison, & Ali [23] the Kingdom of Saudi Arabia found that community pharmacist in the country possesses a significant amount of knowledge to meet the demands for practice in pharmaceutical settings. The practice of counselling is, however, one of the least explored duties of community pharmacists in contemporary Saudi Arabian pharmaceutical literature [24]. In this study, the pharmacist demonstrated effectiveness in counselling, follow up activities, and the practice of vitamins and nutritional supplement dispensing.

One of the primary characteristics of the consumers of vitamins and nutrients is their information-seeking behavior. The results of the study established that a significant population of consumers of vitamins and nutritional supplements were interested in obtaining more information about the nature of the products being consumed. The data was distributed between very good 27.36% and good 39.70% indicating that over 75% of the consumers attending community pharmacist are interested in obtaining data about the nature of vitamins and nutritional supplements dispensed. This suggested that community pharmacist should be aware of the various brands, supplements, and vitamins available in the market, the working mechanism and the side effects [25],[26]. Previous studies [27],[28] have shown that community pharmacists dispensing vitamins and supplements should be aware of the drugs interactions with vitamins and the various supplement products, the side effects of the products [29]. A study conducted by Song et al. [30] found that there was a significant correlation between the health condition being treated and the nature of vitamins and supplements being used. Srikanth et al. [31] found that different groups consume vitamins and nutritional supplements for different reasons. As such, community pharmacists should have the right knowledge of vitamins and nutritional supplements in order to match the demands of the consumers.

In relation to the practice of information acquisition, Williamson et al. [9] found that most community pharmacists obtained information from the internet, pharmacy journals, specific governments websites. The widespread use of smartphones has made it possible for both consumers and community pharmacist to have a wide range of information resources.

## 5. Limitations of the study

While all the necessary precautions were taken to improve the reliability and validity of the samples collected, several challenges could have affected the interpretations and conclusions arrived in this study. The primary limitation of this study was its entire reliance on self-administered questionnaires for data collection. This method of data collection is known to have a likelihood of response bias. In addition, the use of self-evaluation questions to determine the knowledge of community pharmacist on nutrients and vitamins was also another limitation for the study. There is also the increased chance of recall bias since some of the required information came from a distant past. The presence of a knowledgeable resource of community pharmacists in Saudi Arabian pharmacies can be associated with the amount of investment in training and capacity building in the area of vitamins and nutritional supplements [32],[33].

## 6. Conclusion

Community pharmacists in the Saudi Arabia have adequate knowledge and a positive attitude about vitamins and nutritional supplements. The adequacy of knowledge on a wide range of subjects and positive attitudes towards a number of practice activities has already been established in previous studies [34]–[36] among others. The findings of this study call for emphasis on training to improve pharmacists' communication skills. Moreover, communication play more roles in counseling and enhances community pharmacist practice. Future studies done across all KSA regions including villages are needed to include more female pharmacists.

Impact of findings on practice statements: At the policy level, policymakers will be able to develop policies that foster professional development. At the pharmaceutical level, the research provides an avenue to support attempts for developing regular refresher programs on Vitamins, and Nutrition, deficiencies among community pharmacists.
